# A horizontal gene transfer at the origin of phenylpropanoid metabolism: a key adaptation of plants to land

**DOI:** 10.1186/1745-6150-4-7

**Published:** 2009-02-16

**Authors:** Giovanni Emiliani, Marco Fondi, Renato Fani, Simonetta Gribaldo

**Affiliations:** 1 Department of Environmental and Forestry Sciences and Technologies, University of Florence, via S. Bonaventura, 13, 50145, Florence, Italy; 2 Department of Evolutionary Biology, University of Florence, via Romana 19, 50125, Florence, Italy; 3 Institut Pasteur, Unité de Biologie Moléculaire du gène chez les Extrêmophiles, 25 rue du Docteur Roux, 75724, Paris Cedex 15, France

## Abstract

**Background:**

The pioneering ancestor of land plants that conquered terrestrial habitats around 500 million years ago had to face dramatic stresses including UV radiation, desiccation, and microbial attack. This drove a number of adaptations, among which the emergence of the phenylpropanoid pathway was crucial, leading to essential compounds such as flavonoids and lignin. However, the origin of this specific land plant secondary metabolism has not been clarified.

**Results:**

We have performed an extensive analysis of the taxonomic distribution and phylogeny of Phenylalanine Ammonia Lyase (PAL), which catalyses the first and essential step of the general phenylpropanoid pathway, leading from phenylalanine to p-Coumaric acid and p-Coumaroyl-CoA, the entry points of the flavonoids and lignin routes. We obtained robust evidence that the ancestor of land plants acquired a PAL *via *horizontal gene transfer (HGT) during symbioses with soil bacteria and fungi that are known to have established very early during the first steps of land colonization. This horizontally acquired PAL represented then the basis for further development of the phenylpropanoid pathway and plant radiation on terrestrial environments.

**Conclusion:**

Our results highlight a possible crucial role of HGT from soil bacteria in the path leading to land colonization by plants and their subsequent evolution. The few functional characterizations of sediment/soil bacterial PAL (production of secondary metabolites with powerful antimicrobial activity or production of pigments) suggest that the initial advantage of this horizontally acquired PAL in the ancestor of land plants might have been either defense against an already developed microbial community and/or protection against UV.

**Reviewers:**

This article was reviewed by Purificación López-García, Janet Siefert, and Eugene Koonin.

## Background

The appearance of land plants was a key step towards the development of modern terrestrial ecosystems. Fossil data indicate that the first land plants appeared around 500 million years ago, from a pioneer green algal ancestor probably related to Charales [1,2].

**Figure 1:**
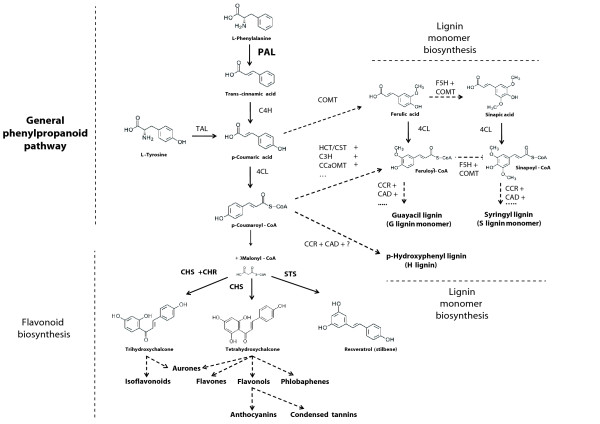
**A schematic representation of phenylpropanoid metabolism**. From the general phenylpropanoid pathway (top left, reactions from L-phenylalanine to p-Coumaroyl-CoA) two separated branches lead to the production of lignin monomers (right) and of flavonoids (bottom). Solid arrows indicate a single step enzymatic reaction, dashed arrows multiple sequential enzymatic reactions. Enzymes are reported with a three letter code: PAL, phenylalanine ammonia lyase; TAL, tyrosine ammonia lyase; C4H, cinnamate 4-hydroxylase; 4CL, 4-coumarate CoA ligase; COMT, caffeic acid/5-hydroxyferulic acid O-methyltransferase; HCT/CST, hydroxycinnamoyl CoA:shikimate/quinate hydroxycinnamoyltransferase; C3H, p-coumaroyl shikimate/quinate 3-hydroxylase; CCoAOMT, caffeoyl CoA O-methyltransferase; CCR, (hydroxy)cinnamoyl CoA reductase; CAD, (hydroxy)cinnamyl alcohol dehydrogenase; F5H ferulate 5-hydroxylase; CHS, chalcone synthase; STS, stilbene synthase.

Early terrestrial environments were harsh. The ancestor of land plants that conquered emerged lands had to face important stresses including desiccation, UV radiation (not anymore shielded by water), as well as attack by already diversified microbial soil communities [1,3]. This drove a number of key adaptations, including the emergence of specialized secondary metabolic pathways. Among them, the phenylpropanoid pathway was crucial. It is in fact a ubiquitous and specific trait of land plants, and provides vital compounds such as lignin -essential for vascularization (xylem) and stem rigidity out of water-, and flavonoids -essential for reproductive biology (flower and fruit colors), protection against UV (pigments) and microbial attack (phytoalexins), and plant-microbe interaction (flavonoids) [4,5]. Three steps constituting the general phenylpropanoid pathway provide the precursors for the flavonoid and lignin branches (Figure 1). Phenylalanine ammonia-lyase (PAL) transforms phenylalanine into trans-cinnamic acid, which leads to p-coumaric acid by the action of cinnamic acid 4-hydrolase (CH4), which is then transformed into p-coumaroyl-CoA by p-coumaroyl:CoA ligase (4CL) (Figure 1). Either p-coumaric acid and p-coumaroyl-CoA can enter the lignin monomer pathway, while p-coumaroyl-CoA is the precursor of the flavonoid pathway (Figure 1). Lignin monomer and flavonoid biosynthesis then involve complex highly branched pathways (Figure 1)[4,6].

The initial physiological advantage of phenolic compounds is not clear. In fact, flavonoids are not thought to have been immediately effective as UV protection before the emergence of complex structures allowing for their accumulation in large quantities, and it has been proposed that they were initially used as internal signaling molecules [7]. Lignin-like polymers have been identified in the cell walls of the charalean alga *Nitella *and in bryophytes (mosses, liverworts, and hornworts), early branching lineages of land plants that do not harbor a developed vascular system such as that found in Tracheophytes (Ferns, Gymnosperms and Angiosperms) [8]. Because these lignin monomers in non vascular plants do not fulfill structural functions it has been proposed that may principally serve as a defense mechanism against microorganisms or UV radiation [8]. To date, there is no evidence for the presence of a full phenylpropanoid metabolism in organisms other than land plants, although some bacteria and fungi harbor homologues of a few enzymes of the pathway [9,10].

The phenylpropanoid pathway likely evolved progressively in land plants by the recruitment of enzymes from the primary metabolism (for a recent review see 4). However, the origin of PAL was a key event, since it provided the initial step from which the rest of the pathway was assembled. Indeed, PAL is a key regulator of the phenylpropanoid pathway [11] and any inhibition of PAL blocks the whole pathway. Probably due to its essentiality, land plants harbor multiple copies of PAL [5] and no complete null mutant is available in the literature. De novo synthesis of PAL is induced in response to different stress stimuli such as UV irradiation, pathogenic attack, low levels of nitrogen, phosphate, or iron [6]. Although PAL enzymes have been extensively characterized in all land plants lineages, including the early emerging bryophytes (mosses, liverworts, and hornworts), their distribution in other organisms is limited. PAL are known to be present in fungi, in particular Basidiomicetes yeasts such as *Rhodotorula*, but also Ascomycetes such as *Aspergillus *and *Neurospora*, where they participate to the catabolism of phenylalanine as a source of carbon and nitrogen [12-14].

The PAL of some plants and fungi also harbor a tyrosine ammonia lyase (TAL) activity that is responsible for the synthesis of p-coumaric acid directly from tyrosine, which in turn leads to the production of p-coumaroyl-CoA [4] (Figure 1). PAL enzymes have been functionally characterized from a few sediment/soil bacteria such as *Streptomyces maritimus *(Actinobacteria), where PAL is required to supply cinnamic acid for the production of benzoyl-CoA, the starter molecule for the biosynthesis of the bacteriostatic agent enterocin [15], and *Photorhabdus luminescens *(γ-Proteobacteria), where PAL is essential for the production of the powerful stilbene antibiotic through yet unknown intermediate steps [16,17]. More recently, PAL have also been identified and structurally characterized in two Cyanobacteria belonging to the order Nostocales, where they are involved in a pathway whose end product is yet unknown [18]. From functional studies, it has been proposed that these cyanobacterial PAL might represent an evolutionary intermediate towards plants PAL [18]. PAL homologues with TAL activity have also been identified in some bacteria such as the Actinobacterium *Saccharotrix espanaensis*, where they are used to produce the antibiotic saccharomicin [19], and in purple phototrophic a-Proteobacteria such as *Rhodobacter*, where they are involved in the synthesis of the chromophore of their photoactive yellow protein photoreceptor [20].

PAL is homologous to histidine ammonia lyase (HAL), which is involved in the catabolism of histidine and is widespread in prokaryotes and eukaryotes [21,22]. It has been proposed that "PAL developed from HAL when fungi and plants diverged from the other kingdoms" [4]. However, the current view of eukaryotic evolution based on phylogenetic analyses indicates that fungi and plants do not share an exclusive ancestor [23,24]. In fact, Fungi are more related to Animals than to land plants. Moreover, land plants belong to the phylum Plantae, which also includes Glaucocystophytes, red algae, and green algae [23,24].

Given the clear importance of PAL in the emergence of the phenylpropanoid pathway and adaptation of plants to land, we sought to get more insight into the origin of this enzyme by carrying out an extensive search of PAL/TAL/HAL homologues in current sequence databases and by analyzing their phylogeny.

## Results

**Figure 2:**
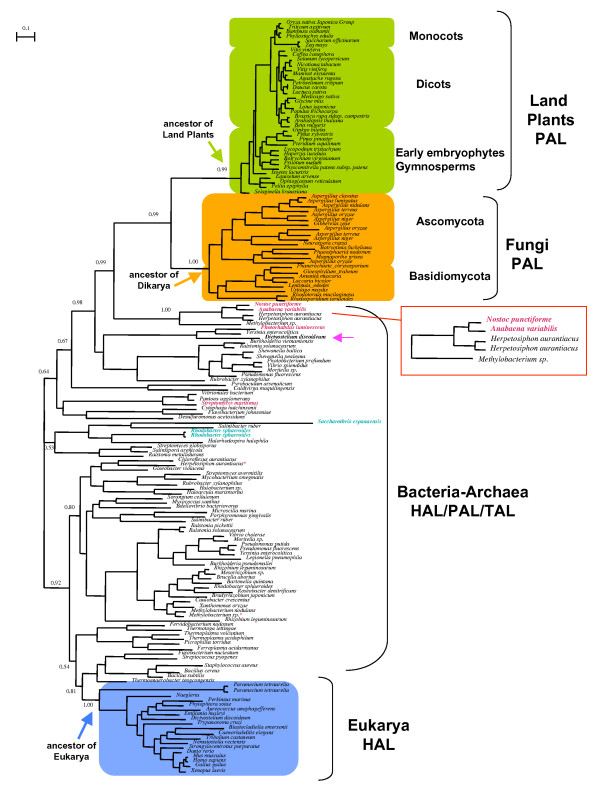
**Phylogeny of PAL/TAL/HAL**. Unrooted bayesian tree of a representative sampling of PAL/TAL/HAL homologues. Characterized bacterial PALs are shown in red font, while characterized bacterial TALs are shown in blue font. Although it is difficult to decide where the root lies, it is clear that eukaryotic HAL (blue square) and fungi/land plants PAL (orange and green squares, respectively) have distinct origins. Moreover, taxonomic distribution of HAL and PAL orthologues indicates that the ancestor of eukaryotes harbored a HAL (blue arrow) while a PAL was introduced by HGT in the ancestor of Dikarya fungi (orange arrow) and the ancestor of land plants (green arrow). The source of this HGT is likely in a group of sediment/soil bacteria including characterized cyanobacterial PAL and uncharacterized sequences from *Methylobacterium sp*. and *Herpetosiphon aurantiacus *(red square). Probable HAL orthologues of *Methylobacterium sp*. and *Herpetosiphon aurantiacus *are indicated by red asterisks. The amoebozoan *Dictyostelium discoideum *appear to have acquired a PAL in the course of a recent HGT from soil bacteria (pink arrow). Numbers at nodes represent posterior probabilities (for clarity only PP relevant for discussion are indicated). The scale bar represents the average number of substitutions per site. The same tree with full accession numbers and PP is provided as Additional file 1. A maximum likelihood analysis gave very similar results and is provided as additional file 2.

Based on preliminary exhaustive phylogenetic analyses, 160 representative sequences were chosen for final tree construction (i.e. a selection of bacterial homologues including all characterized PAL and their closest homologues, all archaeal homologues, a selection of plant and fungi homologues, and all homologues for the remaining eukaryotic phyla, see Methods for details. These sequences are very well conserved and allowed the selection of 369 unambiguously aligned amino acid positions for analysis. The resulting unrooted bayesian tree is shown in Figure 2 (see Additional file 1 and 2 also). The prokaryotic part of the tree is not congruent with species phylogeny, indicating extensive gene duplications, losses, and horizontal gene transfer (HGT) within bacteria as well as between bacteria and archaea, which makes it difficult to retrace the evolutionary history of PAL/TAL/HAL enzymes in prokaryotes. The few characterized bacterial PAL and TAL are not monophyletic (Figure 2, indicated in red and light blue font, respectively), although their close relatives may also be PAL or TAL and it would be interesting to characterize them.

Eukaryotic sequences form two distinct well-supported monophyletic clusters, one including characterized HAL (Figure 2, light blue rectangle, posterior probability PP = 1.00) and the other including characterized PAL (Figure 2, green and orange rectangles, PP = 0.99), separated from each other by bacterial/archaeal homologues. This clearly indicates that eukaryotic PAL and HAL have distinct evolutionary origins, since otherwise eukaryotic PAL should arise from within eukaryotic HAL. HAL homologues are not present in all complete eukaryotic genomes, indicating that catabolism of histidine is not an essential function. However, the cluster of eukaryotic HAL orthologues includes members of major phyla [23,24] such as Alveolates (*Paramecium tetraurelia*, *Perkinsus marinus*), Amoebozoa (*Dictyostelium discoideum*), Haptophytes (*Emiliania huxleyi*), Heterokonts (*Aureococcus anophagefferens*, *Phytophtora soyae*), Excavates (*Naegleria*, *Trypanosoma cruzi*), and Metazoans (Figure 2). This strongly suggests that a HAL was present in the most recent eukaryotic ancestor and the absence of HAL orthologues in some eukaryotic lineages has to be interpreted as a consequence of gene loss. For example, we found no HAL orthologues in any of the fungal lineages for which complete genome sequence data is currently available (i.e. Ascomycota and Basidiomycota, which form the Dikarya [25]), although we retrieved a orthologue in the EST database at NCBI from *Blastocladiella emersonii*, which belongs to the early emerging aquatic lineage Chytridiomycota [25]. This suggests that a HAL orthologue may have been present in the ancestor of Fungi and was secondary lost in Dikarya, but needs confirmation when complete genome sequences will be available from Chytridiomycota and other early emerging fungal lineages. We found no HAL orthologues in any member of the phylum Plantae for which complete genome data is available (i.e. the red algae *Cyanidioschyzon merolae*, the green algae *Chlamydomonas *and *Ostreococcus*, and the Angiosperms *Oryza sativa *and *Arabidopsis thaliana*) [23,24], indicating an early gene loss in this phylum. Intriguingly, *D. discoideum *harbors two additional homologues: one is very divergent and could not be included in the analysis, while the other lies outside of the eukaryotic HAL cluster and close to the characterized PAL of *P. luminescens *(Figure 2, indicated by a pink arrow) and may represent a recent acquisition by HGT. It would be interesting to investigate the role of this putative PAL homologue in *Dictyostelium*, in particular to verify whether it also has an antimicrobial defense role in this soil-dwelling eukaryote, which has been recently suggested to harbor a rudimentary immune system [26].

In contrast to the wide distribution of eukaryotic HAL orthologues, the eukaryotic PAL cluster contains exclusively orthologues from plants and fungi but no other eukaryotic lineage, and these form two well-supported monophyletic sister groups (Figure 2, green and orange rectangles PP = 0.99 and 1.00, respectively). However, the plant PAL cluster includes only members from land plants (including all early emerging lineages such as mosses, hornworts, and liverworts [1,2]), but we found no orthologues in available genomic data from the red and green algae lineages, which branch prior to the divergence of land plants within the phylum Plantae [23,24]. The monophyly of plants PAL orthologues strongly indicates that they have a single origin and derive from a gene that was already present in the ancestor of land plants [27] (Figure 2). Concerning Fungi, the PAL cluster contains the few characterized fungal enzymes (*Amanita*, *Rhodotorula*, *Aspergillus*) and is thus likely that the other orthologues have also PAL activity, although more functional data is needed to verify this. We found PAL orthologues in all complete genomes that are currently available (i.e. exclusively from Dikarya), to the exception of the late emerging lineages Saccharomycotina, Schizosaccharomycetes, and *Cryptococcus *[25], indicating secondary gene losses. We found no PAL orthologues in available genomic data of the fungal lineages Chytridiomycota and Zygomycota, which branch prior to the divergence of Dikarya in the phylogeny of the phylum Fungi [25]. This may indicate absence of a PAL coding gene in these lineages, although this needs to be verified when complete genome data becomes available. The monophyly of the fungal PAL orthologues strongly indicates that they have a single origin and derive from a gene that was already present at least in the ancestor of Dikarya (Figure 2), and possibly earlier.

**Figure 3:**
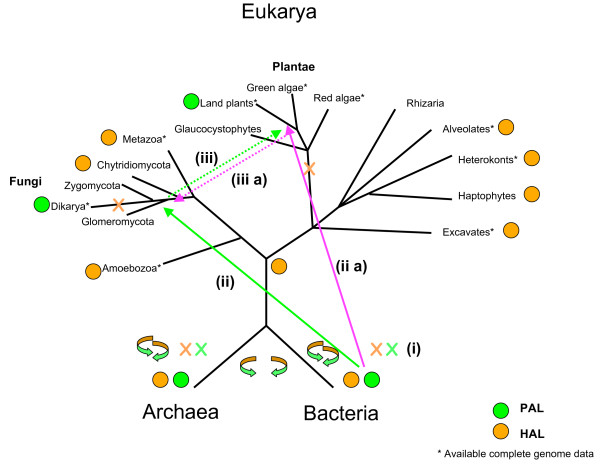
**An evolutionary scenario for the origin of plant PAL**. A HAL coding gene (orange circle) was present in the most recent eukaryotic ancestor, based on its presence in all major eukaryotic supergroups for which sequence data is available (indicated by an asterisk), and it was lost in the ancestor of Dikarya Fungi and in the ancestor of the phylum Plantae (orange crosses). In contrast, the origin of eukaryotic PAL is more recent: (1) origin of PAL in a bacterium (green circle), (2) HGT to fungi -Dikarya or possibly earlier (solid green arrow), (3) HGT from fungi to an ancestor of land plants (dashed green arrow). Alternatively: (1) origin of PAL in a soil bacterium (green circle), (2a) HGT to an ancestor of land plants (solid pink arrow), (3a) HGT from this ancestor to fungi (dashed pink arrow). Extensive HGT of PAL and HAL among and within bacteria and archaea are indicated by double rounded arrows and gene losses by green and orange crosses.

The evolutionary relatedness of PAL orthologues from land plants and fungi clearly indicates a common origin. However, the phylum Plantae does not share an exclusive ancestor with Fungi [23,24], i.e. the most recent common ancestor of these two eukaryotic lineages corresponds the most recent common ancestor of all eukaryotes (Figure 3). Consequently, if a PAL orthologue was present in the ancestor of all eukaryotes, it would have been subsequently lost in all eukaryotic lineages to the exception of land plants and fungi (Figure 3a). A more parsimonious scenario is one where a PAL originated either in the ancestor of land plants or in the ancestor of at least Dikarya fungi and then was transferred *via *HGT between these two phyla (Figure 3b, dotted arrows). Although the prokaryotic part of the tree is blurred by HGT, it is intriguing that a group of bacterial homologues including the two characterized cyanobacterial PAL [18] is robustly supported as sister of the land plants/fungi PAL cluster (PP = 0.99, Figure 2). Again, the heterogeneity of this bacterial group testifies for HGT between its members. In fact, it includes two distantly related sediment/soil bacteria, *Herpetosiphon aurantiacus *(Chloroflexi), and *Methylobacterium *sp. (α-Proteobacteria), a facultative methylotrophic pink pigmented relative of *Rhizobiales *[28,29]. These uncharacterized homologues may also be PAL since these bacteria harbor a second homologue that may be a *bona fide *HAL (indicated by a * symbol in Figure 2).

## Discussion

During early colonization of emerged environments by pioneer land plant ancestors, beneficial associations with fungi and soil bacteria were likely crucial. In particular, it is known that N_2 _fixing cyanobacteria formed symbioses with early fungal lineages (lichen-like or endocytobiotic symbioses, such as those between the glomeromycotan *Geosiphon pyriformis *and the cyanobacterium *Nostoc *[30]) as well as with land plants, and that fungi (Glomeromycota) started arbuscular-mycorrhizal (AM) symbioses with the first land plants [30-34].

The peculiar distribution and phylogeny of plant PAL suggests a plausible scenario for its origin: PAL emerged in bacteria (Figure 3(i)), likely with an antimicrobial role; a member of an early fungal lineage (i.e. at least before the divergence of Dikarya) obtained a PAL *via *HGT from a bacterium (possibly a Nostocale or another soil/sediment bacterium through an early symbiosis [30]) (Figure 3(ii)); this fungal PAL was transferred to an ancestor of land plants *via *an ancient AM symbiosis (Figure 3(iii)), where it paved the way for the development of the phenylpropanoid pathway, and the radiation of plants on terrestrial environments. The fact that land plants PAL do not appear to arise from within fungi PAL (Figure 2) can be explained by the fact that the donor was the ancestor of Dikarya, or by the fact that the donor belonged to a lineage predating the divergence of Dikarya and we either still lack complete genome sequence data from it or the lineage has gone extinct. Important insights to test this evolutionary scenario will be obtained by the future availability of genomic data from early emerging fungal lineages such as Glomeromycota, that possibly emerged before land plants [30] and were most likely the first fungi to form AM type symbioses with them [30,31].

We cannot exclude *a priori *a transfer in a different direction, i.e. from a soil bacterium to an ancestor of land plants *via *an ancient symbiosis (Figure 3(ii a)), then from this to an ancestor of Dikarya fungi (or an earlier branching lineage for which sequence data is not yet available) *via *an ancient AM symbiosis (Figure 3(iii a)). Nevertheless, we wish to stress that land plants PAL are unlikely to be of chloroplastic origin. In fact, since chloroplasts in the phylum Plantae derive from a single primary cyanobacterial endosymbiosis, if land plant PAL had been inherited from the cyanobacterial ancestor of the chloroplast this would imply at least two independent losses of PAL in red and green algal lineages, which postdate the acquisition of the primary chloroplast but emerged prior to the divergence of land plants (Figure 3) [1,2,23,24]. Moreover, only 3 out of the 36 currently available complete cyanobacterial genomes harbor a PAL/HAL homologue (i.e. only *Gloeobacter *in addition to the two Nostocales). PAL is a cytoplasmic enzyme and is not targeted to the chloroplast (we tried to assess the probability of plastid targeting of PAL using the predictions software Predotar V1.03 [35], obtaining no significant results (data non shown). The ancestor of the phylum Plantae likely preceded the ancestor of land plants of many millions of years. If a PAL was transferred by Endosymbiotic Gene Transfer from the cyanobacterial symbiont to the host nucleus in the ancestor of the phylum Plantae, it is not clear why it would have been lost multiple times independently in 2–3 algal lineages, indicating a lack of selective advantage, while being maintained only in the algal line leading to land plants up to around 500 million years ago. Finally, it is possible that the ancestor of land plants and the ancestor of fungi independently acquired their PAL from two different but related bacteria. Importantly, since the gene coding for HAL appears to have been lost early in the phylum Plantae, the phenylpropanoid pathway in land plants could not have been emerged without the acquisition of a PAL homologue by HGT.

## Conclusion

The origin of land plants was a key event in the history of life on our planet since it played a fundamental role in the evolution of modern terrestrial ecosystems. The contribution of bacteria to eukaryotic innovations is considered important, but remains poorly explored. Our results highlight the crucial role of HGT from soil bacteria in the emergence of key metabolic pathways such as that of phenylpropanoids, and therefore in the path leading to land colonization by plants and their subsequent evolution.

Since it is likely that the phenylpropanoid pathway took some time to be fully assembled, it is intriguing to speculate about the original selective advantage to keep a horizontally acquired PAL in the first land plants. The direct products of PAL are cinnamate and p-coumarate. These might have been used as an antimicrobial, such as in some bacteria, and would have played a fundamental role as protection from attack by an already developed microbial soil community. Alternatively (or in combination with an antimicrobial role), they might have provided protection against UV radiation, for example being the precursor of a light capturing pigment such as in modern purple bacteria. Moreover, cinnamate and p-coumarate are the precursors of benzoic acid and salicylic acid, which are known defense compounds [36,37] Finally, it is known that coumarins have appetite suppressing properties, suggesting that an initial role for PAL may have been to provide defense against grazing animals.

It would be interesting to know if fungi also use PAL for these purposes, and what are the corresponding mechanisms for UV shielding and antimicrobial defense in the green algae that are known to colonize soil habitats. To answer these questions, it will be important to investigate further the distribution of PAL enzymes in both bacteria and fungi, which may be more widespread than currently thought, as well as their role in still largely unexplored secondary metabolisms.

## Methods

Exhaustive Blast searches were carried out by using different HAL and PAL sequences as seeds on the non-redundant sequence database and on the EST database at NCBI [38], on ongoing eukaryotic genomes at the DOE Joint Genome Institute [39], at the Broad Institute [40], and at the *Cyanidioschyzon merolae *Genome Project web service [41].

Based on exhaustive preliminary phylogenetic analyses, 160 representative taxa were chosen for final tree construction. From the global alignment, 369 unambiguously aligned amino acid positions were selected for analysis. Tree reconstruction was performed using the bayesian method implemented in MrBayes [42] with a mixed model of amino acid substitution and a gamma correction (eight discrete categories plus a proportion of invariant sites) to take into account among-site rate variations. MrBayes was run with four chains for 1 million generations and trees were sampled every 100 generations. To construct the consensus tree, the first 1500 trees were discarded as "burnin.". Maximum likelihood analysis of the same dataset was carried out by using Phyml [43], with a WAG model of amino acid substitution, including a gamma law with 4 categories to take into account differences in evolutionary rates at sites, and an estimated proportion of invariable sites.

## Abbreviations

PAL: phenylalanine ammonia lyase; TAL: tyrosine ammonia lyase; HAL: histidine ammonia lyase; HGT: horizontal gene transfer; CH4: cinnamic acid 4-hydrolase; 4CL: p-coumaroyl:CoA ligase.

## Competing interests

The authors declare that they have no competing interests.

## Authors' contributions

GE, MF, RF conceived the study, GE MF and SG performed the analyses and all authors drafted the manuscript. All authors read and approved the final manuscript.

## Reviewers' comments

### Reviewer's report 1

#### Purificación López-García

This article presents an extensive molecular phylogenetic analysis of phenylalanine ammonia lyase (PAL, many of which also use tyrosine as substrate), the enzyme catalyzing the first step of the phenylpropanoid pathway leading, in plants and some fungi, to the synthesis of flavonoid secondary metabolites and lignin monomers. The study includes also the related enzyme histidine ammonia lyase (HAL), widespread in the three domains of life. Since land plants and dikaryotic fungi PAL form two sister monophyletic clades clearly distinct from eukaryotic HAL and from their prokaryotic homologues, it is proposed that PAL was transferred horizontally from bacteria to land plants or to fungi and, subsequently, from land plants to fungi or viceversa. This is an interesting observation, well supported by the phylogenetic analysis presented, that leads the authors to hypothesize a key role of this enzyme for the adaptation of plants to land.

I have two major comments. First, the hypothesis that a horizontal gene transfer of PAL to the land plant ancestor is at the origin of the phenylpropanoid metabolism and of their adaptation to terrestrial ecosystems is appealing. However, a single enzyme does not make a pathway and, in the absence of data about the remaining genes involved in phenylpropanoid metabolism, this idea remains hypothetical. In this sense, the title of the article appears too conclusive (*A horizontal gene transfer at the origin of phenylpropanoid metabolism: a key adaptation of plants to land*). Have the authors tried to make preliminary phylogenetic analyses for other genes in the pathway or, at least, do they have an idea about their phylogenetic distribution? It would be interesting to compare the distribution of enzymes involved in flavonoid and lignin monomer biosynthesis with that of PAL.

*AU: We clarified the text to explain that the whole pathway is a specificity of land plants, although a few bacteria and fungi harbor homologues of some enzymes of the pathway. The assembly of the pathway in land plants likely occurred stepwise by the recruitment of preexisting enzymes from other metabolic routes. Although it will be surely interesting to investigate further how this occurred, we now stress in a more clear way that we addressed specifically the very origin of the pathway, which could not have occurred without the acquisition of PAL, since this enzyme performs the first and essential step. Moreover, we precise that such HGT of PAL was essential, since a HAL homologue was likely lost early in the phylum Plantae and therefore land plants could not have assembled the pathway by recruiting a preexisting HAL. Even if the other genes of the pathway were also acquired by HGT, this would have occurred either simultaneously or after the acquisition of PAL. For this reason, our title appears to us justified*.

*Nevertheless, we have performed preliminary analysis of the two enzymes following PAL in the general phenylpropanoid pathway (C4H and 4CL) and these are large gene families that do not appear to show a pattern similar to PAL, supporting the idea that they were recruited from preexisting pathways and strengthening the importance of the HGT of PAL*.

My second comment relates to the primary selective advantage attributed to the acquisition of PAL from bacteria, which might have been the production of antimicrobial or pigmented metabolites that would allow the successful competition of land plants/fungi in soils or protection against UV light. Again, the idea is attractive but, to prove it, would require as a preliminary step to show that the whole flavonoid biosynthesis pathway emerged prior to that of lignin monomer biosynthesis.

If the latter appeared first, one could propose instead that the advantage of acquiring this pathway was to increase stiffness and developing the ability to construct rigid structures, an essential property of land plants and some stages of many fungal life cycles. Perhaps the authors can consider this possibility or discuss why they think it is unlikely. In addition, green algae, which also colonize soil surfaces, have also to compete with other members of the microbial community and to protect themselves from UV. They might have preferred to keep their own, non PAL-derived, protective systems against microbes and UV light.

*AU: We now clarify in the text that early branching land plant lineages harbor the first enzymes of the two main branches of the pathway leading to lignin monomers and flavonoids. Unfortunately, the unavailability of genomic data from earlier lineages (e.g. Charales) prevents understanding for the time being which of the two branches emerged first. We now discuss briefly the production of lignin-like monomers in non-vascular early emerging land plants where these are likely used as defense against either UV or microorganism attack. To our knowledge fungi consume lignin but do not produce it, they construct rigid structures by using chitin*.

*We speculate that the initial selective advantage of PAL that would have lead to the fixation of the HGT may have had to do with the use of its direct products, cinnamic acid and p-coumarate, both involved in antimicrobial or anti UV functions in bacteria and possibly fungi. Moreover, cinnamate and p-coumarate are the precursors of benzoic acid and salicylic acid, which are known defense compounds*.

*The remark on green algae colonizing soil habitats is very interesting. We now discuss it in the text*.

Alternatively, the authors might wish to consider the possibility that flavonoid synthesis did not confer a particular efficient protection against microorganisms, but against metazoan grazers, which constitute indeed the major threat for land plants.

*AU: interesting point, although we do not address specifically the origin of flavonoid production (see above), coumarins have appetite suppressing properties, suggesting its widespread occurrence in plants, especially grasses, is because of its effect of reducing the impact of grazing animals. Thus an immediate advantage of PAL (TAL) might have also been defense against grazers. We now mention it in the text*.

### Reviewer's report 2

#### Eugene Koonin

This straightforward phylogenetic study of Phenylalanine Ammonia Lyase (PAL), the first committed enzyme of the phenylpropanoid pathway, reveals the monophyly of PALs from land plants and dikaryal fungi, with this eukaryotic branch embedded with a highly diverse bacterial tree. The interpretation of this result favored by the authors is that the ancestor of dikaryal fungi acquired the PAL gene from a soil bacterium and passed the gene to the ancestor of land plants. This conclusion implies a key role of HGT in the land colonization by plants.

I think this study highlights both the huge advantages and the considerable headaches that are associated with having numerous genome sequences from all walks of life. The conclusion made by the authors is, of course, interesting and plausible but it is by no means the only one that is possible to make from the tree shown in Figure 2. The main problem, as with most scenarios that involve HGT, is that we do not know the relative likelihoods of HGT and gene loss (but we do know that gene loss in many eukaryotic lineages was extensive). Therefore, arguments for HGT are doomed to remain mostly qualitative and often less than conclusive. With this in mind, potential alternative scenarios for PAL include (but are not necessarily limited to): i) presence in the last common ancestor of the extant eukaryotes with subsequent loss in all lineages (with sequenced genomes) except for land plants and dikaryal fungi; the authors briefly discuss this possibility and dismiss it as unlikely, and generally, one tends to agree (the number of required losses is quite large) but just how unlikely this possibility is, is still hard to tell;

*AU It is indeed hard to tell, but we think that one HGT is a much more parsimonious scenario than massive independent losses in all eukaryotes apart from land plants and fungi. We now explain it more clearly in the text*.

ii) HGT from the chloroplast to the common ancestor of all Plantae, with subsequent loss in algae, followed by HGT to dikaryal fungi; in the manuscript, this scenario is also dismissed as a highly unlikely one but, in this case, I am not sure I agree as the bacterial sister group of the eukaryotic PALs does include some cyanobacteria, and a loss of the gene in 2–3 algal lineages is not unlikely;

AU: at least two reasons make us think that this scenario is unlikely:

*First, even if Nostoc is considered the extant cyanobacterium most similar to the first photosynthetic endosymbiont, only three cyanobacteria over 36 complete genomes harbor PAL/HAL homologues. No chloroplastic genomes harbor a PAL nor a HAL homologue. Furthermore, PAL is a cytoplasmic enzyme and is not targeted to the chloroplast (we tried to assess the probability of plastid targeting of PAL using the predictions software Predotar V1.03, obtaining a not significant result)*.

*Second, the ancestor of the phylum Plantae likely preceded the ancestor of land plants of many millions of years. If a PAL was transferred by Endosymbiotic Gene Transfer from the cyanobacterial symbiont to the host nucleus in the ancestor of the phylum Plantae, it is not clear why it would have been lost multiple times independently in 2–3 algal lineages, indicating a lack of selective advantage, whereas it would have been maintained only in the algal line leading to land plants up to around 500 million years ago*.

*We therefore think that a pal EGT from the cyanobacterial endosymbiont to the host nucleus, although it cannot be excluded a priori, is not a scenario more supported than the one that we propose*.

iii) independent HGTs from related (soil) bacterial to plants and fungi – a possibility that is not discussed in the manuscript but that, as far as I see, cannot be ruled out.

*AU: We included this possibility in the text*.

The above alternatives to the authors' conclusion do not invalidate the work but it must be admitted that, e.g., the chloroplast scenario is less surprising than the one presented by the authors, so much so that the advisability of dedicating a special papers to the origin of PAL in plants and fungi could be questioned. My disappointment with the manuscript is that the authors do not investigate the phylogeny of other enzymes of the phenylpropanoid pathway. Had this been done and had a coherent pattern been discovered, the conclusions could be much more convincing and exciting. If, on the other hand, such a coherent pattern does not exist, this also would be notable indicating that, like many other systems, this key pathway is a patchwork of genes of different origins. I understand, of course, that such a complete phylogenetic analysis requires a considerable amount of extra work, so the authors might prefer to highlight the PAL analysis separately, but I still think that a more comprehensive paper will be of greater value.

*AU: As we explained in our answer to referee 1, we now clarified better in the text that in this report we wished to focus on the very first step in the origin of the pathway that was key to its further assembly. How the pathway was then assembled is surely an interesting question but we feel not directly relevant to our hypothesis. Since without the acquisition of PAL the pathway could not have been be assembled, in particular because of the absence of a preexisting HAL homologue from which a PAL may have been derived, we reckon that our analysis is not incomplete*.

*Indeed, as the referee points out, it would be more exciting not seeing the same pattern for the other genes, and this is what appears from preliminary analysis (see answer to referee 1)*.

At a more technical level, I think that it is highly desirable to also include result from a maximum likelihood analysis to buttress those obtained with the superoptimistic MrBayes. With just one family to analyze, this will not take too much effort.

*AU: this analysis was in fact already done and gave very similar results and statistical support, we now mention it in the text and included the tree as supplementary material 2*.

### Reviewer's report 3

#### Janet Siefert

It's a beginning insight into land plant colonization. I think that other reviewers might have some issues with the argument being based on this one enzyme. I have to admit I did wonder myself about other key enzymes in the phenylpropanoid pathway. I think to help your cause in this regard, you should make a definition of what you mean by the 'first committed step' when you are speaking of the PAL enzyme. The team does a reasonably good job of speculating why the ancestor to land plants might have acquired this gene and it's beneficial use. In figure 2... you need a little bit more information on the methodology for this tree.. it is of course drawn as if it is rooted, is it? This time element to this tree that helps to support your argument presented, is stronger if it is.

*AU: the referee is right, we added some clarifying comments in the legend to *figure 2.

## Supplementary Material

Additional file 1 **Unrooted bayesian tree of Figure **2** with full accession numbers and posterior probabilities.** Click here for file

Additional file 2 **Unrooted ML tree of the same dataset.** Numbers at nodes represent non-parametric bootstrap values caluclated on 100 bootstrapped samples of the original alignment calculated by Phyml (for clarity, not all are shown). For both trees, when no accession number is indicated, the corresponding sequence was retrieved from either the EST database at NCBI or from ongoing genome projects at JGI. EST_chimera indicates chimeric sequences obtained from two different EST sources of the same species. Click here for file
