# Supplementary material for: A horizontal gene transfer at the origin of phenylpropanoid metabolism: a key adaptation of plants to land
Source: Biol Direct. 2009 Feb 16;4:7. doi: 10.1186/1745-6150-4-7 (PMC2657906; doi:10.1186/1745-6150-4-7)
Supplement: Additional file 1 — Unrooted bayesian tree of Figure 2 with full accession numbers and posterior probabilities. [file 1745-6150-4-7-S1.ppt]

## Slide 1
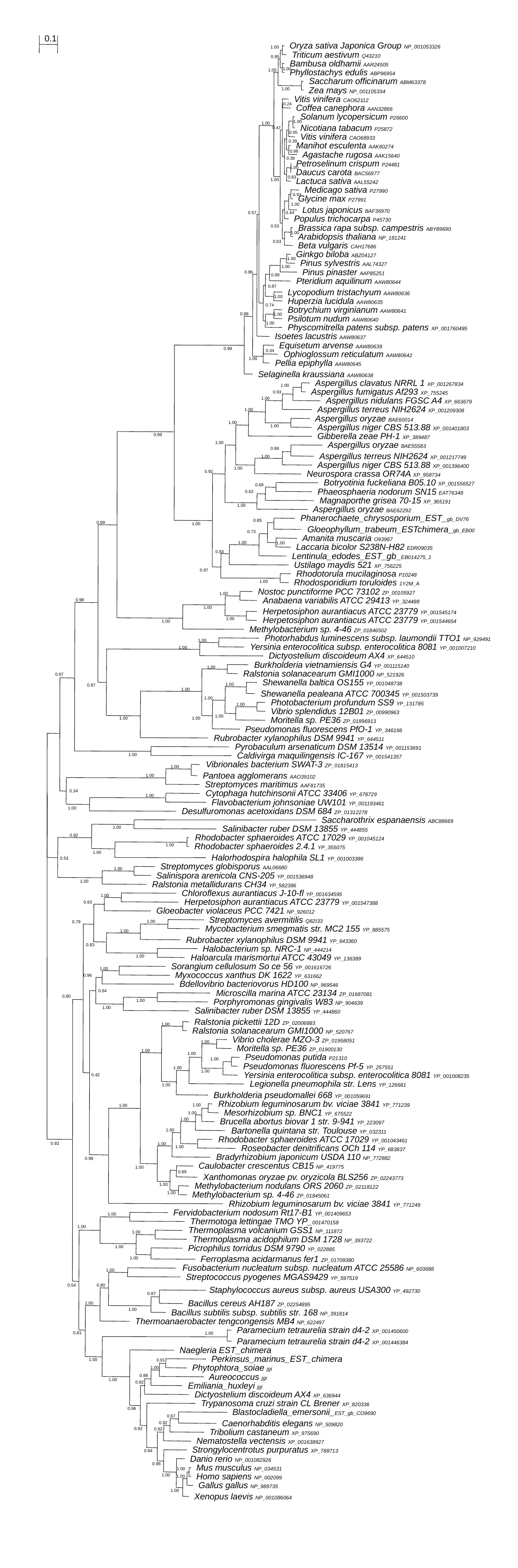

0.1
Oryza sativa Japonica Group NP_001053326
Triticum aestivum Q43210
Bambusa oldhamii AAR24505
Phyllostachys edulis ABP96954
Saccharum officinarum ABM63378
Zea mays NP_001105334
Vitis vinifera CAO62112
Coffea canephora AAN32866
Solanum lycopersicum P26600
Nicotiana tabacum P25872
Vitis vinifera CAO68933
Manihot esculenta AAK60274
Agastache rugosa AAK15640
Petroselinum crispum P24481
Daucus carota BAC56977
Lactuca sativa AAL55242
Medicago sativa P27990
Glycine max P27991
Lotus japonicus BAF36970
Populus trichocarpa P45730
Brassica rapa subsp. campestris ABY89690
Arabidopsis thaliana NP_181241
Beta vulgaris CAH17686
Ginkgo biloba ABZ04127
Pinus sylvestris AAL74327
Pinus pinaster AAP85251
Pteridium aquilinum AAW80644
Lycopodium tristachyum AAW80636
Huperzia lucidula AAW80635
Botrychium virginianum AAW80641
Psilotum nudum AAW80640
Physcomitrella patens subsp. patens XP_001760495
Isoetes lacustris AAW80637
Equisetum arvense AAW80639
Ophioglossum reticulatum AAW80642
Pellia epiphylla AAW80645
Selaginella kraussiana AAW80638
Aspergillus clavatus NRRL 1 XP_001267834
Aspergillus fumigatus Af293 XP_755245
Aspergillus nidulans FGSC A4 XP_663679
Aspergillus terreus NIH2624 XP_001209308
Aspergillus oryzae BAE60014
Aspergillus niger CBS 513.88 XP_001401803
Gibberella zeae PH-1 XP_389487
Aspergillus oryzae BAE55583
Aspergillus terreus NIH2624 XP_001217749
Aspergillus niger CBS 513.88 XP_001396400
Neurospora crassa OR74A XP_958734
Botryotinia fuckeliana B05.10 XP_001556527
Phaeosphaeria nodorum SN15 EAT76348
Magnaporthe grisea 70-15 XP_365191
Aspergillus oryzae BAE62292
Phanerochaete_chrysosporium_EST_gb_DV76
Gloeophyllum_trabeum_ESTchimera_gb_EB00
Amanita muscaria O93967
Laccaria bicolor S238N-H82 EDR09035
Lentinula_edodes_EST_gb_EB014275_1
Ustilago maydis 521 XP_756225
Rhodotorula mucilaginosa P10248
Rhodosporidium toruloides 1Y2M_A
Nostoc punctiforme PCC 73102 ZP_00105927
Anabaena variabilis ATCC 29413 YP_324488
Herpetosiphon aurantiacus ATCC 23779 YP_001545174
Herpetosiphon aurantiacus ATCC 23779 YP_001544654
Methylobacterium sp. 4-46 ZP_01846502
Photorhabdus luminescens subsp. laumondii TTO1 NP_929491
Yersinia enterocolitica subsp. enterocolitica 8081 YP_001007210
Dictyostelium discoideum AX4 XP_644510
Burkholderia vietnamiensis G4 YP_001115140
Ralstonia solanacearum GMI1000 NP_521926
Shewanella baltica OS155 YP_001048738
Shewanella pealeana ATCC 700345 YP_001503739
Photobacterium profundum SS9 YP_131785
Vibrio splendidus 12B01 ZP_00990963
Moritella sp. PE36 ZP_01896913
Pseudomonas fluorescens PfO-1 YP_346166
Rubrobacter xylanophilus DSM 9941 YP_644511
Pyrobaculum arsenaticum DSM 13514 YP_001153691
Caldivirga maquilingensis IC-167 YP_001541357
Vibrionales bacterium SWAT-3 ZP_01815413
Pantoea agglomerans AAO39102
Streptomyces maritimus AAF81735
Cytophaga hutchinsonii ATCC 33406 YP_678729
Flavobacterium johnsoniae UW101 YP_001193461
Desulfuromonas acetoxidans DSM 684 ZP_01312278
Saccharothrix espanaensis ABC88669
Salinibacter ruber DSM 13855 YP_444855
Rhodobacter sphaeroides ATCC 17029 YP_001045124
Rhodobacter sphaeroides 2.4.1 YP_355075
Halorhodospira halophila SL1 YP_001003386
Streptomyces globisporus AAL06680
Salinispora arenicola CNS-205 YP_001536948
Ralstonia metallidurans CH34 YP_582386
Chloroflexus aurantiacus J-10-fl YP_001634595
Herpetosiphon aurantiacus ATCC 23779 YP_001547388
Gloeobacter violaceus PCC 7421 NP_926012
Streptomyces avermitilis Q82I33
Mycobacterium smegmatis str. MC2 155 YP_885575
Rubrobacter xylanophilus DSM 9941 YP_643360
Halobacterium sp. NRC-1 NP_444214
Haloarcula marismortui ATCC 43049 YP_136389
Sorangium cellulosum So ce 56 YP_001616726
Myxococcus xanthus DK 1622 YP_631662
Bdellovibrio bacteriovorus HD100 NP_969546
Microscilla marina ATCC 23134 ZP_01687081
Porphyromonas gingivalis W83 NP_904639
Salinibacter ruber DSM 13855 YP_444860
Ralstonia pickettii 12D ZP_02006883
Ralstonia solanacearum GMI1000 NP_520767
Vibrio cholerae MZO-3 ZP_01958051
Moritella sp. PE36 ZP_01900130
Pseudomonas putida P21310
Pseudomonas fluorescens Pf-5 YP_257551
Yersinia enterocolitica subsp. enterocolitica 8081 YP_001008235
Legionella pneumophila str. Lens YP_126681
Burkholderia pseudomallei 668 YP_001059691
Rhizobium leguminosarum bv. viciae 3841 YP_771239
Mesorhizobium sp. BNC1 YP_675522
Brucella abortus biovar 1 str. 9-941 YP_223097
Bartonella quintana str. Toulouse YP_032311
Rhodobacter sphaeroides ATCC 17029 YP_001043461
Roseobacter denitrificans OCh 114 YP_683837
Bradyrhizobium japonicum USDA 110 NP_772882
Caulobacter crescentus CB15 NP_419775
Xanthomonas oryzae pv. oryzicola BLS256 ZP_02243773
Methylobacterium nodulans ORS 2060 ZP_02118122
Methylobacterium sp. 4-46 ZP_01845061
Rhizobium leguminosarum bv. viciae 3841 YP_771249
Fervidobacterium nodosum Rt17-B1 YP_001409653
Thermotoga lettingae TMO YP_001470158
Thermoplasma volcanium GSS1 NP_111872
Thermoplasma acidophilum DSM 1728 NP_393722
Picrophilus torridus DSM 9790 YP_022885
Ferroplasma acidarmanus fer1 ZP_01709380
Fusobacterium nucleatum subsp. nucleatum ATCC 25586 NP_603688
Streptococcus pyogenes MGAS9429 YP_597519
Staphylococcus aureus subsp. aureus USA300 YP_492730
Bacillus cereus AH187 ZP_02254895
Bacillus subtilis subsp. subtilis str. 168 NP_391814
Thermoanaerobacter tengcongensis MB4 NP_622497
Paramecium tetraurelia strain d4-2 XP_001450600
Paramecium tetraurelia strain d4-2 XP_001446384
Naegleria EST_chimera
Perkinsus_marinus_EST_chimera
Phytophtora_soiae jgi
Aureococcus jgi
Emiliania_huxleyi jgi
Dictyostelium discoideum AX4 XP_636944
Trypanosoma cruzi strain CL Brener XP_820336
Blastocladiella_emersonii_EST_gb_CO9690
Caenorhabditis elegans NP_509820
Tribolium castaneum XP_975690
Nematostella vectensis XP_001638927
Strongylocentrotus purpuratus XP_788713
Danio rerio NP_001082926
Mus musculus NP_034531
Homo sapiens NP_002099
Gallus gallus NP_989735
Xenopus laevis NP_001086064
1.00
0.95
1.00
1.00
1.00
0.24
1.00
1.00
0.47
0.55
0.39
0.98
0.39
1.00
0.83
1.00
0.93
1.00
0.57
0.44
0.53
1.00
0.63
1.00
1.00
0.86
0.89
0.87
1.00
0.74
0.88
1.00
1.00
0.99
0.34
1.00
1.00
0.93
1.00
1.00
1.00
1.00
0.99
1.00
0.99
1.00
1.00
0.92
0.69
0.62
1.00
0.85
0.99
1.00
0.73
1.00
1.00
0.83
0.97
1.00
1.00
0.98
1.00
1.00
1.00
1.00
1.00
1.00
0.67
1.00
0.87
1.00
1.00
1.00
1.00
1.00
1.00
1.00
1.00
1.00
0.34
1.00
1.00
1.00
0.92
1.00
1.00
0.53
1.00
1.00
1.00
0.93
1.00
0.79
1.00
0.83
1.00
1.00
0.96
0.64
0.80
1.00
1.00
1.00
1.00
1.00
1.00
1.00
1.00
1.00
0.42
1.00
1.00
1.00
1.00
1.00
1.00
0.92
1.00
1.00
0.98
1.00
0.99
1.00
1.00
1.00
1.00
1.00
1.00
1.00
1.00
0.54
0.80
0.87
1.00
1.00
0.81
1.00
1.00
0.91
1.00
0.88
1.00
0.82
0.96
0.67
0.92
0.92
0.92
0.94
0.96
1.00
1.00
1.00
1.00
